# Ten simple rules for the computational modeling of behavioral data

**DOI:** 10.7554/eLife.49547

**Published:** 2019-11-26

**Authors:** Robert C Wilson, Anne GE Collins

**Affiliations:** 1 Department of PsychologyUniversity of ArizonaTucsonUnited States; 2 Cognitive Science ProgramUniversity of ArizonaTucsonUnited States; 3 Department of PsychologyUniversity of California, BerkeleyBerkeleyUnited States; 4 Helen Wills Neuroscience InstituteUniversity of California, BerkeleyBerkeleyUnited States; University of OxfordUnited Kingdom

**Keywords:** computational modeling, model fitting, validation, reproducibility

## Abstract

Computational modeling of behavior has revolutionized psychology and neuroscience. By fitting models to experimental data we can probe the algorithms underlying behavior, find neural correlates of computational variables and better understand the effects of drugs, illness and interventions. But with great power comes great responsibility. Here, we offer ten simple rules to ensure that computational modeling is used with care and yields meaningful insights. In particular, we present a beginner-friendly, pragmatic and details-oriented introduction on how to relate models to data. What, exactly, can a model tell us about the mind? To answer this, we apply our rules to the simplest modeling techniques most accessible to beginning modelers and illustrate them with examples and code available online. However, most rules apply to more advanced techniques. Our hope is that by following our guidelines, researchers will avoid many pitfalls and unleash the power of computational modeling on their own data.

## What is computational modeling of behavioral data?

The goal of computational modeling in behavioral science is to use precise mathematical models to make better sense of behavioral data. The behavioral data most often come in the form of choices, but can also be reaction times, eye movements, or other easily observable behaviors, and even neural data. The models come in the form of mathematical equations that link the experimentally observable variables (e.g. stimuli, outcomes, past experiences) to behavior in the immediate future. In this sense, computational models instantiate different ‘algorithmic hypotheses’ about how behavior is generated.

Exactly what it means to ‘make sense’ of behavioral data is, to some extent, a matter of taste that will vary according to the researcher’s goals (Kording et al., 2018). In some cases, a simple model that can explain broad qualitative features of the data is enough. In other cases, more detailed models that make quantitative predictions are required (Breiman, 2001). The exact form of the models, and exactly what we do with them, is limited only by our imaginations, but four uses dominate the literature: simulation, parameter estimation, model comparison, and latent variable inference.

**Simulation** involves running the model with particular parameter settings to generate ‘fake’ behavioral data. These simulated data can then be analyzed in much the same way as one would analyze real data, to make precise, falsifiable predictions about qualitative and quantitative patterns in the data. Simulation is a way to make theoretical predictions more precise and testable. (Some examples include Cohen et al., 1990; Collins and Frank, 2014; Rescorla and Wagner, 1972; Farashahi et al., 2017; Montague et al., 1996; Abbott et al., 2015; Lee and Webb, 2005).
**Parameter estimation** involves finding the set of parameter values that best account for real behavioral data for a given model. These parameters can be used as a succinct summary of a given data set (Ratcliff, 1978; Wilson et al., 2013; Daw et al., 2011; Donkin et al., 2016), for investigating individual differences (Frank et al., 2007; Starns and Ratcliff, 2010; Collins and Frank, 2012; Gillan et al., 2016; Somerville et al., 2017; Nilsson et al., 2011) and for quantifying the effects of interventions such as drugs, lesions, illness, or experimental conditions (Frank et al., 2004; Lorains et al., 2014; Dowd et al., 2016; Zajkowski et al., 2017; Warren et al., 2017; Wimmer et al., 2018; van Ravenzwaaij et al., 2011).
**Model comparison** involves trying to compute which of a set of possible models best describes the behavioral data, as a way to understand which mechanisms are more likely to underlie behavior. This is especially useful when the different models make similar qualitative predictions but differ quantitatively (Wilson and Niv, 2011; Daw et al., 2011; Collins and Frank, 2012; Collins and Frank, 2012; Fischer and Ullsperger, 2013; Steyvers et al., 2009; Haaf and Rouder, 2017; Donkin et al., 2014).
**Latent variable inference** involves using the model to compute the values of hidden variables (for example values of different choices) that are not immediately observable in the behavioral data, but which the theory assumes are important for the computations occurring in the brain. Latent variable inference is especially useful in neuroimaging where it is used to help search for the neural correlates of the model (O'Doherty et al., 2007; Wilson and Niv, 2015; Donoso et al., 2014; Cohen et al., 2017), but also for electroencephalogram (EEG), electrocorticography (ECOG), electrophysiology and pupillometry among many other data sources (O'Reilly et al., 2013; Collins and Frank, 2018; Samejima et al., 2005; Cavanagh et al., 2014; Nassar et al., 2012).

Each of these uses has its strengths and weaknesses, and each of them can be mishandled in a number of ways, causing us to draw wrong and misleading conclusions (Nassar and Frank, 2016; Palminteri et al., 2017). Here we present a beginner-friendly, pragmatic, practical and details-oriented introduction (complete with example code available at [code]) on how to relate models to data and how to avoid many potential modeling mistakes. Our goal for this paper is to go beyond the mere mechanics of implementing models — as important as those mechanics are — and instead focus on the harder question of how to figure out what, exactly, a model is telling us about the mind. For this reason, we focus primarily on the simplest modeling techniques most accessible to beginning modelers, but almost all of our points apply more generally and readers interested in more advanced modeling techniques should consult the many excellent tutorials, didactic examples, and books on the topic (Busemeyer and Diederich, 2010; Daw, 2011; Daw and Tobler, 2014; Heathcote et al., 2015; Huys, 2017; Turner et al., 2013; Vandekerckhove et al., 2015; Wagenmakers and Farrell, 2004; Rigoux et al., 2014; Nilsson et al., 2011; Farrell and Lewandowsky, 2018; Lee et al., 2019).

**Figure 1.**
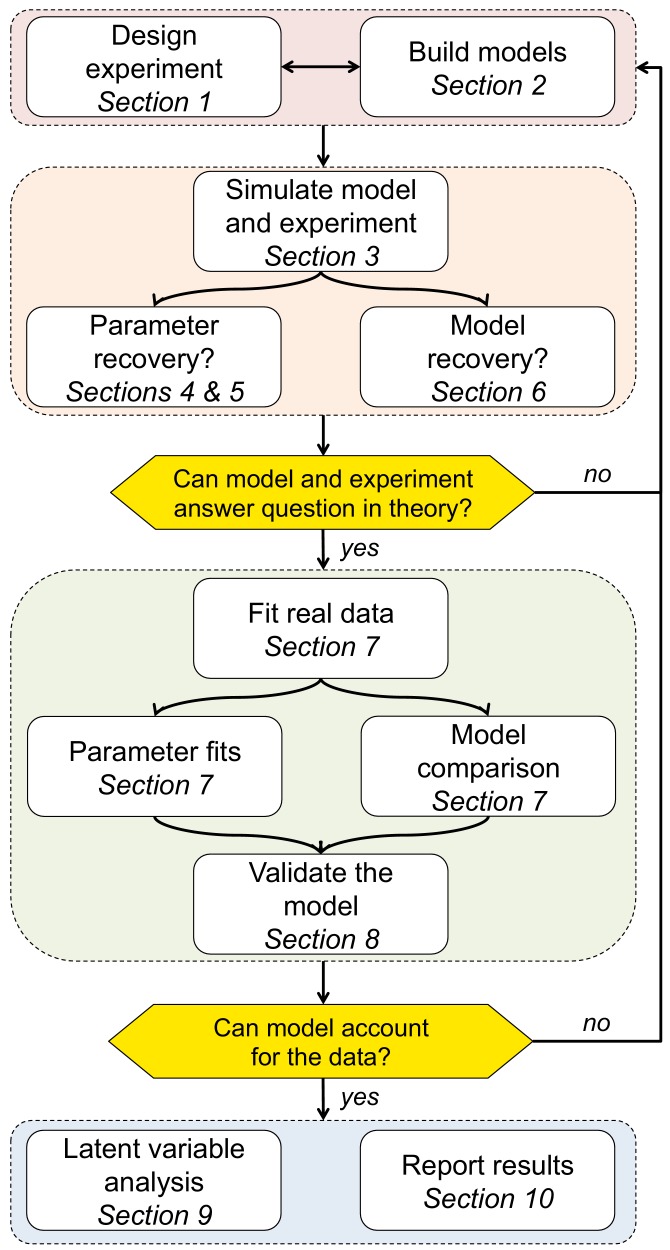
Schematic of the 10 rules and how they translate into a process for using computational modeling to better understand behavior.

For clarity of exposure, we chose to make all of the examples in this paper reflect a single narrow domain - reinforcement learning models applied to choice data (Sutton and Barto, 2018). We chose this domain for a few reasons. (1) Modeling is particularly popular in the field of learning. Indeed, this field benefits from modeling particularly because of the nature of the behavioral data: trials are dependent on all past history and thus unique, making classic data analysis with aggregation across conditions less successful. (2) The sequential dependency of trials in learning contexts can lead to technical challenges when fitting models that are absent in non-learning contexts. However, the same techniques are widely and successfully applied to other observable behavior, such as reaction times (Ratcliff and Rouder, 1998; Viejo et al., 2015; Ballard and McClure, 2019; Wiecki et al., 2013), and to other domains, including but not limited to perception (Sims, 2018), perceptual decision-making (Ratcliff and Rouder, 1998; Drugowitsch et al., 2016; Findling et al., 2018), economic decision-making (van Ravenzwaaij et al., 2011; Nilsson et al., 2011), visual short-term memory (Donkin et al., 2016; Donkin et al., 2014; Nassar et al., 2018), long-term memory (Batchelder and Riefer, 1990), category learning (Lee and Webb, 2005), executive functions (Haaf and Rouder, 2017; Jahfari et al., 2019), and so on. Thus, our hope is that, regardless of the techniques you use or the domain you model, by following these 10 simple steps (Figure 1), you will be able to minimize your modeling mishaps and unleash the power of computational modeling on your own behavioral data!

## Design a good experiment!

Computational modeling is a powerful technique, but it can never replace good experimental design. Modeling attempts to capture how information is manipulated behind the scenes to produce the behavior; thus it is fundamentally limited by the behavioral data, which is itself fundamentally limited by the experimental protocol. A researcher studying face perception would not attempt to fit Prospect Theory to a face perception task; and a researcher studying the differential effects of gain and loss would not do it in a gambling task with only gains. Although obvious in these simple cases, the question becomes more difficult as the complexity of the model increases: is a given learning protocol rich enough to allow the identification of dynamic changes in learning rate, of working memory or episodic memory contributions to learning, or of reward range adaptation? Often, the answer to these questions will be ‘no’ unless the protocol has been deliberately designed to provide this power.

So, how should you go about designing a good experiment with computational modeling in mind? While this process will always be something of an art form, we suggest that you ask yourself the following questions in order to optimize your experimental design:

### What scientific question are you asking?

Although this sounds obvious, it is easy to get sucked into an experimental design without ever asking the most basic questions about your goals. What cognitive process are you targeting? What aspect of behavior are you trying to capture? What hypotheses are you trying to pick apart? For example, you may be trying to identify how working memory contributes to learning or how behavioral variability can be used to explore. Keeping your scientific goals in mind when you design the task can save much time later on.

### Does your experiment engage the targeted processes?

This may be a difficult question to answer, and it may require expert knowledge or piloting. However, you need to know that the experimental design actually engages the processes that you are trying to model.

### Will signatures of the targeted processes be evident from the simple statistics of the data?

In addition to engaging the processes of interest, the best experiments make these processes identifiable in classical analyses of the behavioral data (Palminteri et al., 2017). For example, if you are investigating working memory contributions to learning, you may look for a signature of load on behavior by constructing an experimental design that varies load, to increase chances of probing working memory’s role in learning. Seeing signs of the computations of interest in simple analyses of behavior builds confidence that the modeling process will actually work. In our experience, computational modeling is rarely informative when there is no evidence of an effect in model-independent analyses of behavior.

To answer these questions, it is important to have a clear theoretical hypothesis of what phenomenon is to be modeled. In fact, although designing a good experiment is the first step, it goes hand-in-hand with designing a good model, and the two steps should ideally be done in parallel.

### But what if I’m not an experimentalist?

Computational modeling is hard and many of the best modelers are specialists who never run experiments of their own. Instead these researchers test their models against published findings, publicly available datasets, or even, if they are lucky, unpublished data from their experimental colleagues. Such specialist modelers might feel that they can safely ignore this first point about experimental design and instead focus on explaining the data they can get. We strongly urge them not to. Instead we urge these specialist modelers to always be considering better ways in which their models could be tested. Such experimental thinking helps you to be more concrete in your ideas and to think about how your model might apply outside of the context for which it was designed. In addition, thinking experimentally — and even better talking with experimentalists — forces you to engage with behavior as it actually is rather than as you would like it to be, which in turn can lead to new insights. Finally, by proposing concrete experimental designs, it is easier to convince your experimental colleagues to actually test your ideas, which is surely the goal if we are to move the field forward.

## An illustrative example: the multi-armed bandit task

The ten rules in this paper are quite general, but we will illustrate many of our points using simple examples from our own field of reinforcement learning. Code for implementing all of these examples is available on GitHub (https://github.com/AnneCollins/TenSimpleRulesModeling) (Collins and Wilson, 2019; copy archived at https://github.com/elifesciences-publications/TenSimpleRulesModeling). The goal of these example studies is to understand how people learn to maximize their rewards in a case where the most rewarding choice is initially unknown.

More specifically, we consider the case in which a participant makes a series of T choices between K slot machines, or ‘one-armed bandits’, to try to maximize their earnings. If played on trial t, each slot machine, k, pays out a reward, $r_{t}$, which is one with reward probability, $\mu_{t}^{k}$, and otherwise 0. The reward probabilities are different for each slot machine and are initially unknown to the subject. In the simplest version of the task, the reward probabilities are fixed over time.

The three **experimental parameters** of this task are: the number of trials, T, the number of slot machines, K, and the reward probabilities of the different options, $\mu_{t}^{k}$, which may or may not change over time. The settings of these parameters will be important for determining exactly what information we can extract from the experiment. In this example, we will assume that T=1000, K=2, and that the reward probabilities are $\mu_{t}^{1}=0.2$ for slot machine 1 and $\mu_{t}^{2}=0.8$ for slot machine 2.

## Design good models

Just as bad experiments can limit our ability to test different hypotheses, bad models – quite literally the mathematical embodiment of our hypotheses – can further limit the conclusions we can draw (Donkin et al., 2014). This point is especially important if we are designing new models, but even well-established computational models can be problematic in some cases (Broomell and Bhatia, 2014; Nilsson et al., 2011).

Critical to the design of the model is a clear understanding of your reason for modeling. Are you interested in a descriptive model that succinctly summarizes, but perhaps does not explain, behavioral data? A mechanistic model to tie behavior to the brain? Or an elegant mathematical model to illustrate a concept? As shown in an excellent article by Kording and colleagues (Kording et al., 2018), computational modelers have a wide variety of goals for their models, and understanding your own motivations is a great place to start.

More pragmatically, there are a number of different approaches for designing models that have been successfully used in the literature. Perhaps the simplest approach is to use heuristics to find a ‘reasonable’ way to handle information to produce the target behavior. This approach was how the delta rule (see Model three below) was first invented (Rescorla and Wagner, 1972). Another approach is to scour the artificial intelligence, computer science, and applied mathematics literature for algorithms that have been used to solve similar problems for artificial agents. This approach has been fruitfully applied in the field of reinforcement learning (Sutton and Barto, 2018), where algorithms such as Q-learning and temporal difference learning have been related to human and animal behavior and brain function (Watkins and Dayan, 1992; Montague et al., 1996). Another approach is to take a Bayes-optimal perspective, to design algorithms that perform optimally given a model of the environment and the task. Ideal observer models in vision are one example in which this approach has been applied successfully (Geisler, 2011). More generally, Bayes-optimal models can be further pursued by investigating simpler algorithms that approximate the ideal strategy, or by imposing bounded rationality constraints, such as limited computational resources, on ideal observer agents (Courville and Daw, 2008; Nassar et al., 2010; Collins and Frank, 2012; Daw and Courville, 2007; Lieder et al., 2018).

Regardless of the approach (or, better yet, approaches) that you take to design your models, it is important to keep the following points in mind:

### A computational model should be as simple as possible, but no simpler

Einstein’s old edict applies equally to models of the mind as it does to models of physical systems. Simpler, more parsimonious models are easier to fit and easier to interpret and should always be included in the set of models under consideration. Indeed, formal model comparison techniques (described in detail in Appendix 2) include a penalty for overly complex models, which are more likely to overfit the data and generalize poorly, and favor simpler models so long as they can account for the data.

### A computational model should be interpretable (as much as possible)

In the process of developing models that can account for the behavioral data, researchers run the risk of adding components to a model that are not interpretable as a sensible manipulation of information. For example, a negative learning rate is difficult to interpret in the framework of reinforcement learning. Although such uninterpretable models may sometimes improve fits, nonsensical parameter values may indicate that something important is missing from your model, or that a different cognitive process altogether is at play.

### The models should capture *all* the hypotheses that you plan to test

While it is obviously important to design models that can capture your main hypothesis, it is even more important to design models that capture competing hypotheses. Crucially, *competing models should not be strawmen* — they should have a genuine chance of relating to behavior in the task environment, and they should embody a number of reasonable, graded hypotheses. You should of course put equal effort into fitting these models as you do your favored hypothesis. Better yet, you shouldn’t have a favored hypothesis at all — let the data determine which model is the best fit, not your a priori commitment to one model or another.

Box 1. Example: Modeling behavior in the multi-armed bandit task.

We consider five different models of how participants could behave in the multi-armed bandit task.

 **Model 1: Random responding**

In the first model, we assume that participants do not engage with the task at all and simply press buttons at random, perhaps with a bias for one option over the other. Such random behavior is not uncommon in behavioral experiments, especially when participants have no external incentives for performing well. Modeling such behavior can be important if we wish to identify such ‘checked out’ individuals in a quantitative and reproducible manner, either for exclusion or to study the checked-out behavior itself. To model this behavior, we assume that participants choose between the two options randomly, perhaps with some overall bias for one option over the other. This bias is captured with a parameter b (which is between 0 and 1), such that the probability of choosing the two options is

(1) $$p_{t}^{1}=b\;\text{and}\;p_{t}^{2}=1-b$$

Thus, for two bandits, the random responding model has just one free parameter, controlling the overall bias for option 1 over option 2, ${𝜽}_{1}=b$.

 **Model 2: Noisy win-stay-lose-shift**

The win-stay-lose-shift model is one of the simplest models that adapts its behavior according to feedback. Consistent with the name, the model repeats rewarded actions and switches away from unrewarded actions. In the noisy version of the model, the win-stay-lose-shift rule is applied probabilistically, such that the model applies the win-stay-lose-shift rule with probability 1-ϵ, and chooses randomly with probability ϵ. In the two-bandit case, the probability of choosing option k is

(2) $$p_{t}^{k}=\left\{ \begin{array}{cc} 1-\epsilon /2 & \text{if }\left(c_{t-1}=k\text{and }r_{t-1}=1\right)\text{OR }\left(c_{t-1}\neq k\text{and }r_{t-1}=0\right)\\ \epsilon /2 & \text{if }\left(c_{t-1}\neq k\text{and }r_{t-1}=1\right)\text{OR }\left(c_{t-1}=k\text{and }r_{t-1}=0\right) \end{array}\right.$$

where $c_{t}=1,2$ is the choice at trial t, and $r_{t}=0,1$ the reward at trial t. Although more complex to implement, this model still only has one free parameter, the overall level of randomness, ${𝜽}_{2}=\epsilon$.

**Model 3: Rescorla Wagner**

In this model, participants first *learn* the expected value of each slot machine based on the history of previous outcomes and then use these values to make a *decision* about what to do next. A simple model of learning is the Rescorla-Wagner learning rule (Rescorla and Wagner, 1972), whereby the value of option k, $Q_{t}^{k}$ is updated in response to reward $r_{t}$ according to:

(3) $$Q_{t+1}^{k}=Q_{t}^{k}+\alpha \,\left(r_{t}-Q_{t}^{k}\right)$$

where α is the learning rate, which takes a value between 0 and 1 and captures the extent to which the prediction error, $\left(r_{t}-Q_{t}^{k}\right)$, updates the value. For simplicity, we assume that the initial value, $Q_{0}^{k}$, is zero, although it is possible to treat the $Q_{0}^{k}$ as a free parameter of the model.

A simple model of decision making is to assume that participants use the options’ values to guide their decisions, choosing the most valuable option most frequently, but occasionally making ‘mistakes’ (or exploring) by choosing a low-value option. One choice rule with these properties is known as the ‘softmax’ choice rule, which chooses option k with probability

(4) $$p_{t}^{k}=\frac{\exp\left(\beta \,Q_{t}^{k}\right)}{\sum_{i=1}^{K}\exp\left(\beta \,Q_{t}^{i}\right)}$$

where β is the ‘inverse temperature’ parameter that controls the level of stochasticity in the choice, ranging from β=0 for completely random responding and β=∞ for deterministically choosing the highest value option.

Combining the learning (Equation 3) and decision rules (Equation 4) gives a simple model of decision-making in this task with two free parameters: the learning rate, α, and the inverse temperature, β. That is, in our general notation, for this model ${𝜽}_{3}=\left(\alpha ,\beta \right)$.

**Model 4: Choice kernel**

This model tries to capture the tendency for people to repeat their previous actions. In particular, we assume that participants compute a ‘choice kernel,’ $C\,K_{t}^{k}$, for each action, which keeps track of how frequently they have chosen that option in the recent past. This choice kernel updates in much the same way as the values in the Rescorla-Wagner rule, i.e. according to

(5) $$C\,K_{t+1}^{k}=C\,K_{t}^{k}+\alpha_{c}\,\left(a_{t}^{k}-C\,K_{t}^{k}\right)$$

where $a_{t}^{k}=1$ if option k is played on trial t, otherwise $a_{t}^{k}=0$, and $\alpha_{c}$ is the choice-kernel learning rate. For simplicity, we assume that the initial value of the choice kernel is always zero, although, like the initial Q-value in the Rescorla-Wagner model, this could be a parameter of the model. Note that with $\alpha_{c}=1$, this model is very similar to model 2 (win-stay-lose-shift). From there, we assume that each option is chosen according to

(6) $$p_{t}^{k}=\frac{\exp\left(\beta_{c}\,C\,K_{t}^{k}\right)}{\sum_{i=1}^{K}\exp\left(\beta_{c}\,C\,K_{t}^{i}\right)}$$

where $\beta_{c}$ is the inverse temperature associated with the choice kernel.

Combining the choice kernel (Equation 5) with the decision rule (Equation 6) gives a simple model of decision-making in this task with two free parameters: the choice-kernel learning rate, $\alpha_{c}$, and the choice-kernel inverse temperature $\beta_{c}$. That is, in our general notation, for this model ${𝜽}_{4}=\left(\alpha_{c},\beta_{c}\right)$.

 **Model 5: Rescorla Wagner + choice kernel**

Finally, our most complex model mixes the reinforcement learning model with the choice kernel model. In this model, the values update according to Equation 3, while the choice kernel updates according to Equation 5. The terms are then combined to compute the choice probabilities as

$$p_{t}^{k}=\frac{\exp\left(\beta \,Q_{t}^{k}+\beta_{c}\,C\,K_{t}^{k}\right)}{\sum_{i=1}^{K}\exp\left(\beta \,Q_{t}^{i}+\beta_{c}\,C\,K_{t}^{i}\right)}$$

This most complex model has four free parameters, i.e. ${𝜽}_{5}=\left(\alpha ,\beta ,\alpha_{c},\beta_{c}\right)$.

## Simulate, simulate, simulate!

Once you have an experimental design and a set of computational models, a really important step is to create *fake*, or *surrogate* data (Palminteri et al., 2017). That is, you should use the models to simulate the behavior of participants in the experiment, and to observe how behavior changes with different models, different model parameters, and different variants of the experiment. This step will allow you to refine the first two steps: confirming that the experimental design elicits the behaviors assumed to be captured by the computational model. To do this, here are some important steps.

### Define model-independent measures that capture key aspects of the processes you are trying to model

Finding qualitative signatures (and there will often be more than one) of the model is crucial. By studying these measures with simulated data, you will have greater intuition about what is going on when you use the same model-independent measures to analyze real behavior (Daw et al., 2011; Collins and Frank, 2012; Collins and Frank, 2013; Nassar et al., 2018; Lee and Webb, 2005).

### Simulate the model across the range of parameter values

Then, visualize behavior as a function of the parameters. Almost all models have free parameters. Understanding how changes to these parameters affect behavior will help you to better interpret your data and to understand individual differences in fit parameters. For example, in probabilistic reinforcement learning tasks modeled with a simple delta-rule model (Model 3; Equation 3), the learning rate parameter, α, can relate to both the speed of learning and noisiness in asymptotic behavior, as can the inverse temperature parameter, β (in Equation 4), as seen in Box 2—figure 1B.

### Visualize the simulated behavior of different models

This will allow you to verify that behavior is qualitatively different for different models, making their predictions in the experimental setup different (Box 2—figure 1A). If the behavior of different models is *not* qualitatively different, this is a sign that you should try to design a better experiment. Although not always possible, distinguishing between models on the basis of qualitative patterns in the data is always preferable to quantitative model comparison (Navarro, 2019; Palminteri et al., 2017).

More generally, the goal of the simulation process is to clarify how the models and experimental design satisfy your goal of identifying a cognitive process in behavior. If the answer is positive — i.e. the experiment is rich enough to capture the expected behavior, the model’s parameters are interpretable, and competing models make dissociable predictions — you can move on to the next step. Otherwise, you should loop back through these first three sections to make sure that your experimental design and models work well together, and that the model parameters have identifiable effects on the behavior, which is a prerequisite for the fourth step, fitting the parameters (c.f. Figure 1).

Box 2. Example: simulating behavior in the bandit task.

To simulate behavior, we first need to define the parameters of the task. These include the total number of trials, T (=1000 in the example), as well as the number of bandits, K(=2), and the reward probability for each bandit, $\mu^{k}$ (0.2 and 0.8 for bandits 1 and 2, respectively). The experiment parameters, as used in the simulation, should match the actual parameters used in the experiment.

Next we define the parameters of the model. One way to do this is to sample these parameters randomly from prior distributions over each parameter, the exact form of which will vary from model to model. These prior distributions should generally be as broad as possible, but if something is known about the distribution of possible parameter values for a particular model, this is one place to include it.

With the free parameters set, we then proceed with the simulation. First, we simulate the choice on the first trial, $a_{1}$, by assuming that the model chooses option k with probability, $p_{1}^{k}$. Next we simulate the outcome, $r_{1}$, of this choice. In Models 2–5, we use the action and/or outcome to update the choice probabilities for the next trial. Repeating this process for all trials up to t=T completes one simulation. The simulations can then be analyzed in the same way as participants’ data is, ideally with the same code taking different inputs. This process should be repeated several times, with different parameter settings, to get a handle on how the model behaves as a function of its parameters.

To illustrate how one might visualize the simulated results, we look at two model-independent measures that should capture fundamental aspects of learning: the probability of repeating an action, p⁢(stay) (should I change my behavior in response to feedback?), and the probability of choosing the correct option, p⁢(correct) (have I learned?). In Box 2—figure 1A below, we plot p⁢(stay) as a function of the reward on the last trial for each of the models with a particular set of parameters (M1: b=0.5, M2: ϵ=0.05, M3: α=0.1, β=5, M4: $\alpha_{c}=0.1$, $\beta_{c}=3$, M5: α=0.1, β=5, $\alpha_{c}=0.1$, $\beta_{c}=1$). For some models (in particular the win-stay-lose-shift model (Model 2), we expect a strong dependence on past reward, but for others, such as the random responder (Model 1), we expect no dependence. Of course, the exact behavior of each model depends crucially on the parameters used in the simulations and care should be taken to ensure that these simulation parameters are reasonable, perhaps by matching to typical parameter values used in the literature or by constraining to human-like overall performance. Better yet is to simulate behavior across a range of parameter settings to determine how the model-independent measures change with different parameters.

A more thorough exploration of the parameter space for Model 3 is shown in Box 2—figure 1B, where we plot the p⁢(correct) in the first and last 10 trials as a function of the learning rate, α, and softmax parameter, β. Note that the ‘optimal’ learning rate, i.e. the value of α that maximizes p(correct), varies between early and late trials and as a function of the softmax parameter β, where for early trials higher β implies a lower optimal α (Daw et al., 2011).

The question of how to choose the model-independent measures of behavior has no easy answer and calls to the domain knowledge of the specific scientific question that the modeler is attempting to answer. As a rule of thumb, the measures should capture global characteristics (e.g. overall performance) and diagnostic measures that relate to the question of interest, and may visualize different qualitative predictions of different models.

**Box 2—figure 1.**
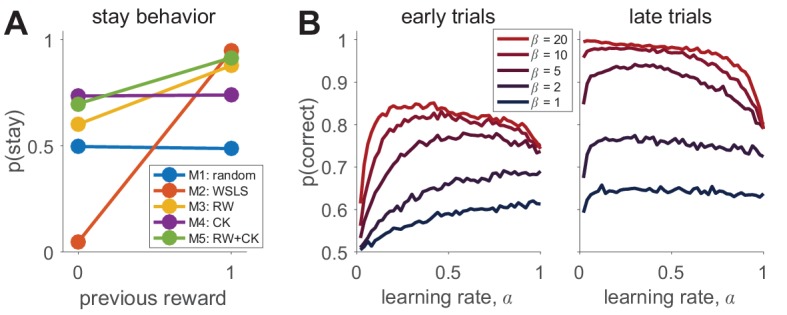
Simulating behavior in the two-armed bandit task. (**A**) Win-stay-lose-shift behavior varies widely between models. (**B**) Model 3 simulations (100 per parameter setting) show how the learning rate and softmax parameters influence two aspects of behavior: early performance (first 10 trials), and late performance (last 10 trials). The left graph shows that learning rate is positively correlated with early performance improvement only for low β values or for very low α values. For high β values, there is a U-shape relationship between learning rate and early speed of learning. The right graph shows that with high β values, high learning rates negatively influence asymptotic behavior. Thus, both parameters interact to influence both the speed of learning and asymptotic performance.

## Fit the parameters

A key component of computational modeling is estimating the values of the parameters that best describe your behavioral data. There are a number of different ways of estimating parameters, but here we focus on the maximum-likelihood approach, although almost all of our points apply to other methods such as Markov Chain Monte Carlo approaches (Lee and Wagenmakers, 2014). Mathematical details, as well as additional discussion of other approaches to model fitting can be found in Appendix 1.

In the maximum likelihood approach to model fitting, our goal is to find the parameter values of model m, ${\hat{𝜽}}_{m}^{M\,L\,E}$, that maximize the likelihood of the data, $d_{1:T}$, given the parameters, $p\,\left(d_{1:T}|{𝜽}_{m},m\right)$. Maximizing the likelihood is equivalent to maximizing the log of the likelihood, $L\,L=\log p\,\left(d_{1:T}|{𝜽}_{m},m\right)$, which is numerically more tractable. (The likelihood is a product of many numbers smaller than 1, which can be rounded to 0 with limited precision computing. By contrast, the log-likelihood is a sum of negative numbers, which is usually tractable and will not be rounded to 0.) A simple mathematical derivation shows that this log-likelihood can be written in terms of the choice probabilities of the individual model as

(8) $$L\,L=\log p\,\left(d_{1:T}|{𝜽}_{m},m\right)=\sum_{t=1}^{T}\log p\,\left(c_{t}|d_{1:t-1},s_{t},{𝜽}_{m},m\right)$$

where $p\,\left(c_{t}|d_{1:t-1},s_{t},{𝜽}_{m},m\right)$ is the probability of each individual choice given the parameters of the model and the information available up to that choice, which is at the heart of the definition of each model (for example in Equations 1-7).

In principle, finding the maximum likelihood parameters is as ‘simple’ as maximizing L⁢L. In practice, of course, finding the maximum of a function is not a trivial process. The simplest approach, a brute force search of the entire parameter space, is occasionally useful, and may help you to understand how different parameters interact (see Box 3—figure 1). However, this approach is unfeasible outside of the simplest cases (e.g. one or two parameters with tight bounds) because of the high computational costs of evaluating the likelihood function at a large number of points.

Fortunately, a number of tools exist for finding local maxima (and minima) of functions quickly using variations on gradient ascent (or descent). For example, Matlab’s fmincon function can use a variety of sophisticated optimization algorithms (e.g. Moré and Sorensen, 1983; Byrd et al., 2000) to find the minimum of a function (and other factors such as the Hessian that can be useful in some situations [Daw, 2011]). So long as one remembers to feed fmincon the *negative* log-likelihood (whose minimum is at the same parameter values as the maximum of the positive log-likelihood), using tools such as fmincon can greatly speed up model fitting. Even here, though, a number of problems can arise when trying to maximize L⁢L that can be reduced by using the tips and tricks described below. Most of the tips come from understanding that optimization algorithms are not foolproof and in particular are subject to numerical constraints. They generalize to other black box optimization functions in other languages, for example the Python scipy.optimize package or the optim function in R.

### Be sure that your initial conditions give finite log-likelihoods

Optimizers such as fmincon require you to specify initial parameter values from which to start the search. Perhaps the simplest way in which the search process can fail is if these initial parameters give log-likelihoods that are not finite numbers (e.g. infinities or NaNs, not a number in Matlab speak). If your fitting procedure fails, this can often be the cause.

### Beware rounding errors, zeros and infinities

More generally, the fitting procedure can go wrong if it encounters infinities or NaNs during the parameter search. This can occur if a choice probability is rounded down to zero, thus making the log of the choice probability -∞. Likewise, if your model involves exponentials (e.g. the softmax choice rule in Equation 4), this can lead to errors whereby the exponential of a very large number is ‘rounded up’ to infinity. One way to avoid these issues is by constraining parameter values to always give finite choice probabilities and log-likelihoods at the boundaries. One way to diagnose these issues is to include checks in the code for valid log-likelihoods.

### Be careful with constraints on parameters

If the constraints are ill chosen, it is possible that the solution will be at the bounds, which is often, but not always, a red flag.

Only include parameters that have an influence on the likelihood. If only two parameters impact the likelihood, but the optimizer attempts to fit three, it will usually find the optimum for the two relevant parameters and a random value for the third; however, it will lead to slower and less efficient fitting.

### Beware local minima!

Finally, a key limitation of optimization algorithms is that they are only guaranteed to find *local* minima, which are not guaranteed to be the *global* minima corresponding to the best fitting parameters. One way to mitigate this issue is to run the fitting procedure multiple times with random initial conditions, recording the best fitting log-likelihood for each run. The best fitting parameters are then the parameters corresponding to the run with the highest log-likelihood. There is no hard-and-fast rule for knowing how many starting points to use in a given situation, besides the fact that more complex models will require more starting points. Thus, this number must be determined empirically in each case. One way to validate the number of starting points is by plotting the best likelihood score as a function of the number of starting points. As the number of initial conditions increases, the best-fitting likelihood (and corresponding the parameters) will improve up to an asymptote close to the true maximum of the function (e.g. Box 3—figure 1).

Box 3. Example: contending with multiple local maxima.

As a real example with local maxima, we consider the case of a simplified version of the mixed reinforcement learning and working memory model from Collins and Frank, 2012. For simplicity, we relegate the details of this model to Appendix 4. To appreciate the example, all one really needs to know is that in its simplest version, this model has two parameters: ρ, which captures the effect of working memory, and α, which captures the learning rate of reinforcement learning. As is seen in Box 3—figure 1 below, this model (combined with an appropriate experiment) gives rise to a log-likelihood surface with multiple local maxima. Depending on the starting point, the optimization procedure can converge to any one of these local maxima, meaning that the ‘maximum’ likelihood fits may not reflect the global maximum likelihood.

To mitigate this concern, a simple and effective approach is to repeat the optimization procedure many times, keeping track of the best fitting log-likelihood and parameters in each case. An approximation to the global maximum is to take the best log-likelihood from this list of fits. The results of this multiple iteration procedure can be summarized by plotting the best log-likelihood as a function of the number of starting points, or similarly, by plotting the distance from the so-far best parameters to the final best parameters as a function of the number of starting points (Box 3—figure 1B). As the number of starting points increases, the best-fitting log-likelihood and parameters will converge to the global maximum. This plot also allows us to judge when we have used enough starting points. Specifically, if the best fitting parameters appear to have reached asymptote, that gives us a good indication that the fit is the best we can do.

**Box 3—figure 1.**
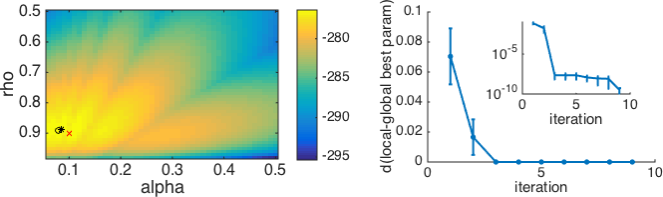
An example with multiple local minima. (**Left**) Log-likelihood surface for a working memory reinforcement learning model with two parameters. In this case, there are several local minima, all of which can be found by the optimization procedure depending on the starting point. Red x, generative parameters; black circle, optimum with brute search method; black *, optimum with fmincon and multiple starting points. (**Right**) Plotting the distance from the best fitting parameters after n iterations to the best fitting parameters after all iterations as a function of the number of starting points n gives a good sense of when the procedure has found the global optimum. The inset shows the same plot on a logarithmic scale for distance, illustrating that there are still very small improvements to be made after the third iteration.

## Check that you can recover your parameters

Before reading too much into the best-fitting parameter values, ${𝜽}_{m}^{M\,L\,E}$, it is important to check whether the fitting procedure gives meaningful parameter values in the best case scenario, -that is, when fitting fake data where the ‘true’ parameter values are known (Nilsson et al., 2011). Such a procedure is known as ‘Parameter Recovery’, and is a crucial part of any model-based analysis.

In principle, the recipe for parameter recovery is quite simple. First, simulate fake data with known parameter values. Next, fit the model to these fake data to try to ‘recover’ the parameters. Finally, compare the recovered parameters to their true values. In a perfect world, the simulated and recovered parameters will be tightly correlated, with no bias. If there is only a weak correlation between the simulated and recovered parameters and/or a significant bias, then this is an indication that there is either a bug in your code (which from our own experience is fairly likely) or the experiment is underpowered to assess this model.

To make the most of your parameter recovery analysis, we suggest the following tips:

### Make sure your simulation parameters are in the right range

An important choice for parameter recovery is the range of simulation parameters that you wish to recover. Some models/experiments only give good parameter recovery for parameters in a particular range — if the simulation parameters are too big or too small, they can be hard to recover. An illustration of this is the softmax parameter, β, where very large β values lead to almost identical behavior in most experiments. Thus parameter recovery may fail for large β values but work well for small β values. Of course, selecting only the range of parameters that *can* be recovered by your model is not necessarily the right choice, especially if the parameter values you obtain when fitting real data are outside of this range! For this reason, we have the following recommendations for choosing simulation parameter values:

1. If you have already fit your data, we recommend matching the range of your simulation parameters to the range of values obtained by your fit.
2. If you have not fit your data but you are using a model that has already been published, match the range of parameters to the range seen in previous studies.
3. Finally, if the model is completely new and the ‘true’ parameter values are unknown, we recommend simulating over as wide a range as possible to get a sense of whether and where parameters can be recovered. You can rely on your exploration of how model parameters affect simulated behavior to predict a range beyond which parameters will not affect behavior much.

Note that it is not necessarily problematic if a model’s parameters are not recoverable in a full parameter space, as long as they are recoverable in the range that matters for real data.

### Plot the correlations between simulated and recovered parameters

While the correlation coefficient between simulated and recovered parameters is a useful number for summarizing parameter recovery, we also strongly recommend that you actually plot the simulated vs recovered parameters. This makes the correlation clear, and also reveals whether the correlation holds in some parameter regimes but not others. It also reveals any existing bias (for example, a tendency to recover higher or lower values in average).

### Make sure the recovery process does not introduce correlations between parameters

In addition to looking at the correlations between simulated and recovered parameters, we also recommend looking at the correlation between the recovered parameters themselves. If the simulation parameters are uncorrelated with one another, correlation between the recovered parameters is an indication that the parameters in the model are trading off against one another (Daw, 2011). Such trade-offs can sometimes be avoided by reparameterizing the model (e.g. Otto et al., 2013) or redesigning the experiment. Sometimes, however, such trade-offs are unavoidable. In these cases, it is crucial to report the trade-off in parameters so that a ‘correlation’ between fit parameter values is not over-interpreted in real data.

A note about parameter differences between different populations or conditions: a growing use of model fitting is to compare parameter values between populations (e.g. schizophrenia patients vs healthy controls [Collins et al., 2014]) or conditions (e.g., transcranial magnetic stimulation to one area or another [Zajkowski et al., 2017]). If your primary interest is a difference like this, then parameter recovery can be used to give an estimate of statistical power. In particular, for a proposed effect size (e.g., on the average difference in one parameter between groups or conditions) you can simulate and recover parameters for the groups or conditions and then perform statistical tests to detect group differences in this simulated data set. The power for this effect size is then the frequency with which the statistical tests detect no effect given that the effect is there.

### Remember that even successful parameter recovery represents a best-case scenario!

What does successful parameter recovery tell you? That data generated by a known model with given parameters can be fit to recover those parameters. This is the best case you could possibly hope for in the model-based analysis and it is unlikely to ever occur as the ‘true’ generative process for behavior — that is, the inner workings of the mind and brain — is likely much more complex than any model you could conceive. There’s no easy answer to this problem. We only advise that you remember to be humble when you present your results!

Box 4. Example: parameter recovery in the reinforcement learning model.

We performed parameter recovery with Model 3, the Rescorla Wagner model, on the two-armed bandit task. As before, we set the means of each bandit at $\mu_{1}=0.2$ and $\mu_{2}=0.8$ and the number of trials at T=1000. We then simulated the actions of the model according to Equations 3 and 4, with learning rate, α, and softmax temperature, β, set according to

(9) α∼U⁢(0,1) and β∼Exp⁢(10)

After simulating the model, we fit the parameters using a maximum likelihood approach to get fit values of learning rate, α, and softmax parameter, β. We then repeated this process 1000 times using new values of α and β each time. The results are plotted in Box 4—figure 1 below. As is clear from this plot, there is fairly good agreement between the simulated and fit parameter values. In addition, we can see that the fit for β is best with a range, 1<β<10, and that outside this range, the correspondence between simulation and fit is not as good. If we further select points where parameter recovery for α is bad (i.e., when $\left|\alpha_{s\,i\,m}-\alpha_{f\,i\,t}\right|>0.25$, grey dots in Box 4—figure 1), we find that parameter recovery for α is worse when β is outside of the range. Depending on the values of β that we obtain by fitting human behavior, this worse correspondence at small and large β values may or may not be problematic. It may be a good idea to use the range of parameters obtained from fitting the real data to test the quality of recovery within the range that matters.

**Box 4—figure 1.**
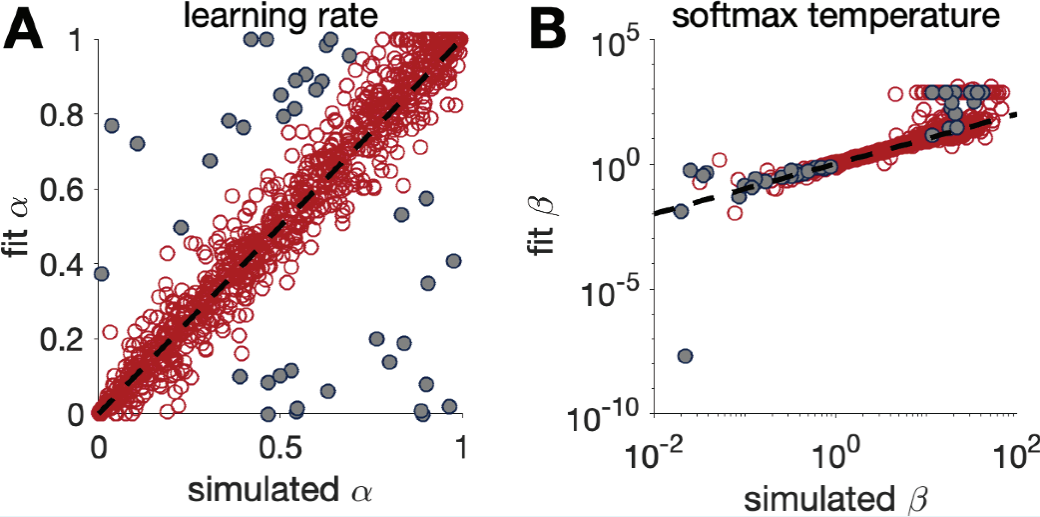
Parameter recovery for the Rescorla Wagner model (model 3) in the bandit task with 1000 trials. Grey dots in both panels correspond to points where parameter recovery for α is bad.

## Can you arbitrate between different models?

In model comparison, our goal is to determine which model, out of a set of possible models, is most likely to have generated the data. There are a number of different ways to make this comparison (summarized in more detail in Appendix 2) that involve different approximations to the Bayesian evidence for each model (e.g., Daw, 2011; Rigoux et al., 2014). Here, we focus on the most common method which is related to the log-likelihood computed in 'Fit the parameters'.

A simplistic approach to model comparison would be to compare the log-likelihoods of each model at the best fitting parameter settings, $p\,\left(d_{1:T}|{\hat{𝜽}}_{m},m\right)$. However, if the data, $d_{1:T}$, used to evaluate the log-likelihood are the same as those used to fit the parameters, then this approach will lead to overfitting, as the model with the most free parameters will almost always fit this ‘training’ data best. As an extreme example, consider the case of a model with one ‘parameter’ per choice, which is the identity of the choice the person actually made. Such a ‘model’ would fit the data perfectly, but would of course tell us nothing about how the choices were actually determined and would make no predictions about what choices would be made in a different setting. Overfitting is a problem in that it decreases the generalizability of the model: it makes it less likely that the conclusions drawn would apply to a different sample.

One way to avoid overfitting is to perform cross-validation: by measuring fit on held-out data, we directly test generalizability. However, this is not always possible for practical reasons (number of samples) or more fundamental ones (dependence between data points). Thus, other methods mitigate the risk of overfitting by approximately accounting for the degrees of freedom in the model. There are several methods for doing this (including penalties for free parameters), which are discussed in more detail in the Appendices. There is a rich theoretical literature debating which method is best (Wagenmakers and Farrell, 2004; Vandekerckhove et al., 2015). Here, we do not position ourselves in this theoretical debate, and instead focus on one of the simplest methods, the Bayes Information Criterion, B⁢I⁢C, which has an explicit penalty for free parameters.

(10) $$B\,I\,C=-2\,\log\hat{L\,L}+k_{m}\,\log\left(T\right)$$

where $\hat{L\,L}$ is the log-likelihood value at the best fitting parameter settings, and $k_{m}$ is the number of parameters in model m. The model with the smallest B⁢I⁢C score is the model that best fits the data. Thus, the positive effect of $k_{m}$ in the last term corresponds to a penalty for models with large numbers of parameters.

While Equation 10 is simple enough to apply in order to find the model that, apparently, best fits your data, it is important to check that your model comparison process gives sensible results for simulated data. Just as parameter fitting should be validated by parameter recovery on simulated data, so model comparison should be validated by model recovery on simulated data.

More specifically, model recovery involves simulating data from all models (with a range of parameter values carefully selected as in the case of parameter recovery) and then fitting that data with all models to determine the extent to which fake data generated from model A is best fit by model A as opposed to model B. This process can be summarized in a confusion matrix (see Box 5—figure 1 below for an example) that quantifies the probability that each model is the best fit to data generated from the other models, that is, p(fit model=B|simulated model=A). In a perfect world, the confusion matrix will be the identity matrix, but in practice, this is not always the case (e.g., Wilson and Niv, 2011).

When computing and interpreting a confusion matrix it is important to keep the following points in mind:

### Compare different methods of model comparison

If the confusion matrix has large off-diagonal components, then you have a problem with model recovery. There are a number of factors that could cause this problem, ranging from a bug in the code to an underpowered experimental design. However, one cause that is worth investigating is whether you are using the wrong method for penalizing free parameters. In particular, different measures penalize parameters in different ways that are ‘correct’ under different assumptions. If your confusion matrix is not diagonal, it may be that the assumptions underlying your measures (e.g. BIC) do not hold for your models, in which case it might be worth trying another metric for model comparison (e.g., AIC [Wagenmakers and Farrell, 2004]; see Appendix 2).

### Be careful with the choice of parameters when computing the confusion matrix

Just as parameter recovery may only be successful in certain parameter regimes, so too can model recovery depend critically on the parameters chosen to simulate the models. In some parameter regimes, two models may lead to very different behavior, but they may be indistinguishable in other parameter regimes (see Box 5—figure 1 below). As with parameter recovery, we believe that the best approach is to match the range of the parameters to the range seen in your data, or to the range that you expect from prior work.

### A note on interpreting the confusion matrix

As described above, and in keeping with standard practice from statistics, the confusion matrix is defined as the probability that data simulated by one model is best fit by another, that is, p⁢(fit model|simulated model). However, when we fit a model to real data, we are usually more interested in making the reverse inference — that is, given that model B fits our data best, which model is most likely to have generated the data? This is equivalent to computing p⁢(simulated model|fit model). Note that this measure, which we term the ‘inversion matrix’ to distinguish it from the confusion matrix, is not the same as the confusion matrix unless model recovery is perfect. Of course, the inversion matrix can be computed from the confusion matrix using Bayes rule (see Appendix 3) and it may be useful to report it in cases where the confusion matrix is not diagonal.

### The elephant in the room with model comparison

As wonderful as it is to find that your model ‘best’ fits the behavioral data, the elephant in the room (or perhaps more correctly *not* in the room) with all model comparison is that it only tells you which of the models you considered fits the data best. In and of itself, this is rather limited information as there are infinitely many other models that you did not consider. This makes it imperative to start with a good set of models that rigorously capture the competing hypotheses (that is, think hard in Step 2). In addition, it will be essential to validate (at least) your winning model (see Step 9) to show how simulating its behavior can generate the patterns seen in the data that you did not explicitly fit, and thus obtain an *absolute* measure of how well your model relates to your data.

Box 5. Example: confusion matrices in the bandit task.

To illustrate model recovery, we simulated the behavior of the five models on the two-armed bandit task. As before, the means were set at $\mu_{1}=0.2$ and $\mu_{2}=0.8$, and the number of trials was set at T=1000. For each simulation, model parameters were sampled randomly for each model. Each simulated data set was then fit to each of the given models to determine which model fit best (according to BIC). This process was repeated 100 times to compute the confusion matrices which are plotted below in Box 5—figure 1A and B.

The difference between these two confusion matrices is in the priors from which the simulation parameters were sampled. In panel A, parameters were sampled from the following priors:

**Table:** 

**Model**	**Priors**
Model 1	b∼U⁢(0,1)
Model 2	ϵ∼U⁢(0,1)
Model 3	α∼U⁢(0,1), β∼Exp⁢(1)
Model 4	$\alpha_{c}∼U\,\left(0,1\right)$, $\beta_{c}∼\text{Exp}\,\left(1\right)$
Model 5	α∼U⁢(0,1), β∼Exp⁢(1), $\alpha_{c}∼U\,\left(0,1\right)$, $\beta_{c}∼\text{Exp}\,\left(1\right)$

In panel B, all of the softmax parameters β and $\beta_{c}$ were increased by 1. This has the effect of reducing the amount of noise in the behavior, which makes the models more easily identifiable and the corresponding confusion matrix more diagonal. The fact that the confusion matrix can be so dependent on the simulating parameter values means that it is crucial to match the simulation parameters to the actual fit parameters as best as possible. Models that are identifiable in one parameter regime may be impossible to distinguish in another!

In addition to the confusion matrices, we also plot the inversion matrices in Box 5—figure 1C and D. These are computed from the confusion matrices using Bayes rule assuming a uniform prior on models (see Appendix 3). These matrices more directly address the question of how to interpret a model comparison result where one model fits a particular subject best.

**Box 5—figure 1.**
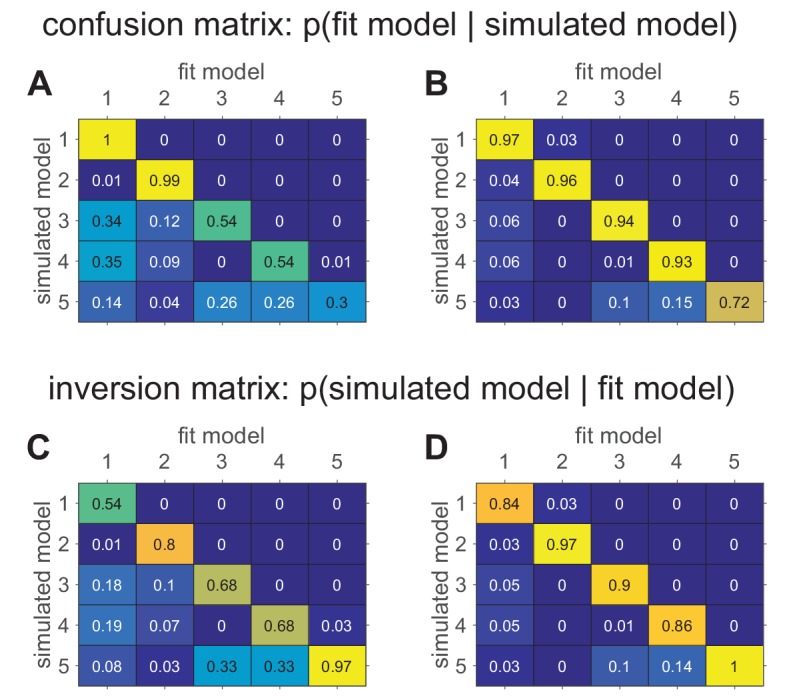
Confusion matrices in the bandit task showing the effect of prior parameter distributions on model recovery. Numbers denote the probability that data generated with model X are best fit by model Y, thus the confusion matrix represents p⁢(fit model|simulated model). (**A**) When there are relatively large amounts of noise in the models (possibility of small values for β and $\beta_{c}$), models 3–5 are hard to distinguish from one another. (**B**) When there is less noise in the models (i.e. minimum value of β and $\beta_{c}$ is 1), the models are much easier to identify. (**C**) The inversion matrix provides easier interpretation of fitting results when the true model is unknown. For example, the confusion matrix indicates that M1 is always perfectly recovered, while M5 is only recovered 30% of the time. By contrast, the inversion matrix shows that if M1 is the best fitting model, our confidence that it generated the data is low (54%), but if M5 is the best fitting model, our confidence that it did generate the data is high (97%). (**D**) Similar results with less noise in simulations.

## Run the experiment and analyze the actual data

Once all the previous steps have been completed, you can finally move on to modeling your empirical data. The first step to complete is of course to analyze the data *without* the model, in the same way that we recommended for model simulations in section 'Simulate, simulate, simulate!' This model-independent analysis is extremely important: you designed the experiment to test specific hypotheses, and constructed models to reflect them. Simulations showed expected patterns of behaviors given those hypotheses. If the model-independent analyses do not show evidence of the expected results, there is almost no point in fitting the model. Instead, you should go back to the beginning, either re-thinking the computational models if the analyses show interesting patterns of behavior, or re-thinking the experimental design or even the scientific question you are trying to answer. In our experience, if there is no model-independent evidence that the processes of interest are engaged, then a model-based analysis is unlikely to uncover evidence for the processes either.

If, however, the behavioral results are promising, the next step is to fit the models developed previously and to perform model comparison. After this step, you should check that the parameter range obtained with the fitting is within a range where parameter and model recovery were good. If the range is outside what you explored with simulations, you should go back over the parameter and model recovery steps to match the empirical parameter range, and thus ensure that the model fitting and model comparison procedures lead to interpretable results.

An important point to remember is that human behavior is always messier than the model, and it is unlikely that the class of models you explored actually contains the ‘real’ model thatgenerated human behavior. At this point, you should consider looping back to Steps 2–5 to improve the models, guided by in depth model-independent analysis of the data.

For example, you may consider modeling ‘unimportant parameters’, representing mechanisms that are of no interest to your scientific question but that might still affect your measures. Modeling these unimportant parameters usually captures variance in the behavior that would otherwise be attributed to noise, and as such, makes for a better estimation of ‘important’ parameters. For example, capturing pre-existing biases (e.g. a preference for left/right choices) in a decision or learning task provides better estimation of the inverse temperature, by avoiding attributing systematic biases to noise, which then affords better estimation of other parameters like the learning rate (this is evident in Box 6—figure 1).

Box 6. Example: improving parameter recovery by modeling unimportant parameters.

To illustrate the effect that ‘unimportant’ parameters (i.e., parameters that represent mechanisms that are of no interest to your scientific question, but may still affect your measures) can have on fitting results, we model the effect of a side bias on parameter recovery in Model 3. In particular, we assume that, in addition to choosing based on learned value, the model also had a side bias, B, that effectively changes the value of the left bandit. That is, in the two-bandit case, the choice probabilities are given by

(11) $$p_{t}^{l\,e\,f\,t}=\frac{1}{1+\exp\left(\beta \,\left(Q_{t}^{r\,i\,g\,h\,t}-Q_{t}^{l\,e\,f\,t}-B\right)\right)}$$

We then simulated behavior with this model for a range of parameter values and fit the model with the original version of model 3, without the bias, and the modified version of model 3, with the bias. In this simulation, agents learn for 10 independent two-armed bandits in successive 50-trial blocks, with μ={0.2,0.8} or μ={0.8,0.2} in different blocks. For simplicity, we assumed that the agent treats each block as independent, and started from the same initial values of $Q_{1}^{r\,i\,g\,h\,t}=Q_{1}^{l\,e\,f\,t}=0.5$.

As can be seen below, including the ‘unimportant’ bias in the fit greatly improves the extent to which both the learning rate, α, and softmax parameter, β, can be recovered.

**Box 6—figure 1.**
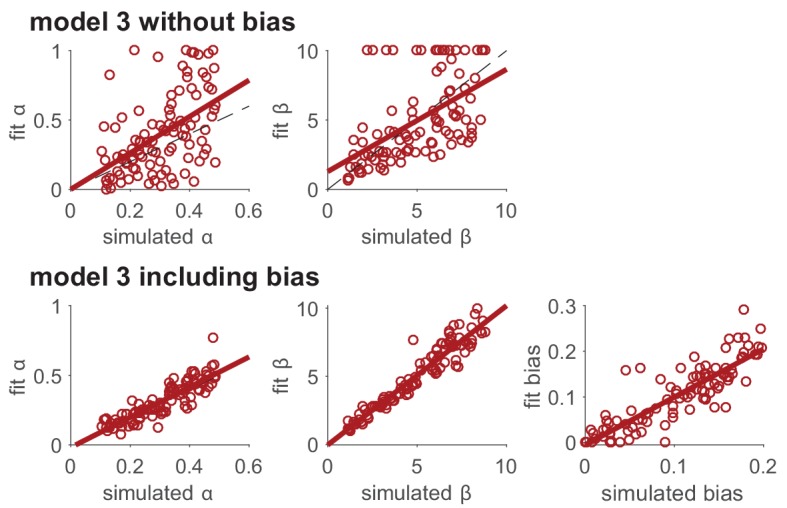
Modeling unimportant parameters provides better estimation of important parameters. The top row shows parameter recovery of the model without the bias term. The bottom row shows much more accurate parameter recovery, for all parameters, when the bias parameter is included in the model fits.

## Validate (at least) the winning model

All the previous steps measure a *relative* goodness of fit. Does model A fit better than model B? However, before interpreting any results from a model, it is essential to ensure that the model actually usefully captures the data in an *absolute* sense. This step is called model validation, and should never be skipped: it is possible to fit a model, get high fit measures, and nevertheless completely miss the essence of the behavior.

One method for model validation is computing the average trial likelihood as an absolute measure of fit. Although this measure has some nice properties — for example, the best possible value is one when the model predicts behavior perfectly — it offers limited value when choices are actually stochastic (which may be the case in many situations; Drugowitsch et al., 2016) or the environment is complex. In these cases, the best possible likelihood per trial is less than 1, but it is not known what the best possible likelihood per trial could be. For this reason, although the likelihood per trial can be a useful tool for model validation (Leong et al., 2017), interpreting it as an absolute measure of model fit is of limited value.

A better method to validate a model is to simulate it with the fit parameter values (Palminteri et al., 2017; Nassar and Frank, 2016; Navarro, 2019), a procedure long performed by statisticians as part of the ‘posterior predictive check’ (Roecker, 1991; Gelman et al., 1996). You should then analyze the simulated data in the same way that you analyzed the empirical data, to verify that all important behavioral effects are qualitatively and quantitatively captured by the simulations with the fit parameters. For example, if you observe a qualitative difference between two conditions empirically, the model should reproduce it. Likewise, if a learning curve reaches a quantitative asymptote of 0.7, simulations shouldn’t reach a vastly different one.

Some researchers analyze the posterior prediction of the model conditioned on the past history, instead of simulated data. In our previous notation, they evaluate the likelihood of choice $c_{t}$ given past data, $d_{1:t-1}$, where the past data includes choices made by the subject, *not* choices made by the model, $p\,\left(c_{t}|d_{1:t-1},s_{t},{𝜽}_{m},m\right)$. In some cases, this approach leads to very similar results to simulations, because simulations sample choices on the basis of a very similar probability, where the past data, $d_{1:t-1}$, include choices made by the *model*. However, it can also be dramatically different if the path of actions sampled by the participant is widely different from the paths likely to be selected by the model (leading to very different past histories).

Palminteri and colleagues (Palminteri et al., 2017) offer a striking example of this effect, where Model A fits better than Model B by any quantitative measure of model comparison, but is completely unable to capture the essence of the behavior. In their example, data are generated with a reinforcement learning agent (which takes the place of the subject) on a reversal learning task (where a choice that was previously good becomes bad, and reciprocally). These data are then fit with either a win-stay lose-shift model (model B), or a simplistic choice kernel model, which assumes that previous choices tend to be repeated (model A). Because of the autocorrelation in the choices made by the reinforcement learning agent, model A, which tends to repeat previous actions, fits better than model B, whose win-stay-lose-shift choices only depend on the action and outcome from the last trial. However, model A is completely insensitive to reward, and thus is unable to generate a reversal behavior when it is simulated with the fit model parameters. Thus, in this case, model A should be discarded, despite a greater quantitative fit. Nevertheless, the fact that the best validating model B captures less variance than model A should serve as a warning that model B is missing crucial components of the data and that a better model probably exists. This should incite the researcher to go back to the drawing board to develop a better model, for example one that combines elements of both models or a different model entirely, and perhaps a better experiment to test it.

More generally, if your validation step fails, you should go back to the drawing board! This may involve looking for a better model, as well as redesigning the task. Be careful interpreting results from a model that is not well validated! Of course, exactly what it means for a model to ‘fail’ the validation step is not well defined: no model is perfect, and there is no rule of thumb to tell us when a model is *good enough*. The most important aspect of validation is for you (and your readers) to be aware of its limitations, and in which ways they may influence any downstream results.

Box 7. Example: model validation where the fit model performs too well.

Most examples of model validation involve a case where a model that fits well performs poorly on the task in simulation. For example, in the Palminteri et al. (2017) example, the choice kernel model cannot perform the task at all because its behavior is completely independent of reward. Here, we offer a different example of failed model validation in which the model performs *better* in simulation than the predicted and observed artificial agent’s behavior. Moreover, this model appears to fit data generated from a different model better than it fits data generated from itself! In this example, we imagine a deterministic stimulus-action learning task in which agents are presented with one of three stimuli ($s_{1}$, $s_{2}$, and $s_{3}$), which instruct them which of three actions ($a_{1}$, $a_{2}$, and $a_{3}$) will be rewarded when chosen. $a_{1}$ is the correct choice for both stimuli $s_{1}$ and $s_{2}$, $a_{3}$ for $s_{3}$, and $a_{2}$ is incorrect for all stimuli.

The two models that we consider are both reinforcement learning agents. The first, a ‘blind’ agent does not see the stimulus at all and learns only about the value of the three different actions, that is $Q\,\left(a_{i}\right)$, regardless of the stimulus. The second, a ‘state-based’ agent, observes the stimulus and learns a value for each action that can be different for each stimulus, that is $Q\,\left(a_{i},s_{i}\right)$. Parameters in the models are set such that the learning curves for the two agents are approximately equal (Box 7—figure 1A). See appendices for details of the models.

We then consider how both models fit behavior simulated by either of these models. In Box 7—figure 1B, we plot the average likelihood with which the state-based model predicts the actual choices of the blind and state-based agents, that is the average $p\left(c_{t}|d_{1:t-1},{𝜽}_{m},m=\text{state-based}\right)$. As is clear from this figure, the state-based model predicts choices from the blind agent with *higher* likelihood than choices from the state-based agent! While counter intuitive, this result does not imply that the state-based model is unable to fit its own behavior. Instead, this result reflects the difference in noise (softmax parameters) between the two agents. The blind RL agent has a low noise parameter, allowing the state-based model to fit it quite well. Conversely, the state-based RL agent has a high noise parameter, meaning that the behavior is harder to predict even when it is fit with the correct model.

That the state-based model captures state-based behavior better than it fits blind behavior is illustrated in Box 7—figure 1C. Here, we plot the simulated learning curves of the state-based model using the parameter values that were fit to either the state-based agent or the blind agent. Although the parameters of the state-based model obtained through the fit to the state-based agent generate a learning curve that is quite similar to that of the agent (compare blue lines in Box 7—figure 1A and C), the state-based fit to the blind agent performs too well (compare yellow lines in Box 7—figure 1A and C).

Thus the model validation step provides support for the state-based model when it is the correct model of behavior, but rules out the state-based model when the generating model was different. The take-away from this example should be that measures of model-fit and model comparison cannot replace a thorough validation step, which can contradict them.

**Box 7—figure 1.**
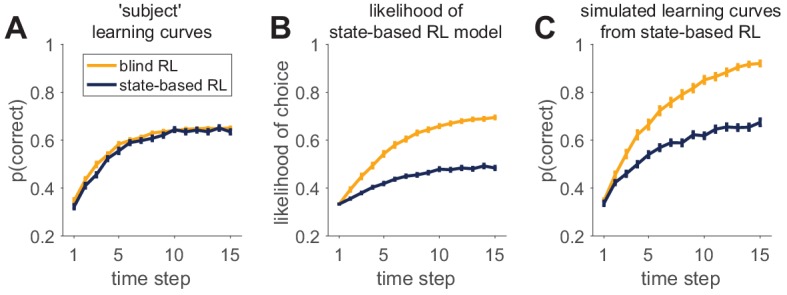
An example of successful and unsuccessful model validation. (**A**) Behavior is simulated by one of two reinforcement learning models (a blind agent and a state-based agent) performing the same learning task. Generative parameters of the two models were set so that the learning curves of the models were approximately equal. (**B**) Likelihood per trial seems to indicate a worse fit for the state-based-simulated data than the blind-simulated data. (**C**) However, validation by model simulations with fit parameters shows that the state-based model captures the data from the state-based agent (compare dark learning curves in panels A and C), but not from the the blind agent (yellow learning curves in panels A and C).

## Analyze the winning model

To minimize risks of p-hacking, model-dependent analyses should only be performed on the winning model, after researchers are satisfied that the model captures the behavior. One particularly powerful application of model-based analysis of behavior involves estimating the *latent* variables in the model. Latent variables are the hidden components of the algorithms underlying the behavior that are not directly observable from the behavior itself. These latent variables shed light on the internal workings of the model and, if we are to take the model seriously, should have some representation in the subjects’ mind and brain (Cohen et al., 2017; O'Doherty et al., 2007).

Extracting latent variables from the model is as simple as simulating the model and recording how the latent variables evolve over time. The parameters of the simulation should be the fit parameters for each subject. In most cases, it is useful to yoke the choices of the model to the choices the participants actually made, thus the latent variables evolve according to the experience participants actually had. This is especially true if the choices can influence what participants see in the future.

Once estimated, the latent variables can be used in much the same way as any other observable variable in the analysis of data. Perhaps the most powerful application comes when combined with physiological data such as pupil dilation, EEG, and fMRI. The simplest of these approaches uses linear regression to test whether physiological variables correlate with the latent variables of interest. Such an approach has led to a number of insights into the neural mechanisms underlying behavior (Nassar et al., 2012; Daw et al., 2011; Donoso et al., 2014; Collins and Frank, 2018; Fischer and Ullsperger, 2013), although, as with any modeling exercise, latent variable analysis should be done with care (Wilson and Niv, 2015).

Other model-dependent analyzes include studying individual differences as captured by fit parameters. Fit parameters can be treated as a dependent variable in continuous analyses (e.g. correlating with age, symptom scales, and so on [Gillan et al., 2016]) or group comparisons (e.g. patients vs. matched controls [Collins et al., 2014]).

## Reporting model-based analyses

Congratulations! You have developed, simulated, and fit your model (and maybe several other competing models) to your data. You have estimated parameters, computed model comparison scores, and validated whether your model can generate realistic-looking behavior. It’s time to start writing! But what exactly should you report in your paper? And how should you report it?

### Model selection

In many modeling papers, a key conclusion from the work is that one model fits the data better than other competing models. To make this point convincingly, we recommend including the following things in your paper, either as main results or in the supplementary material.

#### Model recovery analysis

##### Confusion matrix

Before anyone should believe your model comparison results, you need to demonstrate the ability of your analysis/experiment to distinguish between models under ideal conditions of simulated data. The best way to visualize these results is with a confusion matrix, as outlined in section 'Can you arbitrate between different models'? If the model comparison result is central to your paper, we recommend including the confusion matrix as a figure in the main text. If model comparison is less important, we recommend including it in the supplementary materials.

##### Number of subjects best fit by each model

The simplest way to visualize how well the winning model fits the data is with a histogram showing the number of subjects best fit by each model. Obviously if all subjects are best fit with one model, the story is simple. The more likely scenario is that some subjects will be best fit by other models. Such a result is important to acknowledge in the paper as it may reflect the use of different strategies by different people or that the ‘correct’ model lies somewhere in between the models you have considered.

#### Group level statistics

##### Exceedance probabilities

A more sophisticated and less biased (Piray et al., 2018) way to report model comparison results is by computing the probability that a single model best describes all the data. This is clearly an assumption whose merits should be discussed in your paper. In cases where it is valid, the method of Rigoux et al. (2014) computes these ‘Exceedance Probabilities’, the probability that each model generated all the data. These probabilities can also be reported in histogram or table form.

Model-independent measures of simulated data. The cleanest way to demonstrate the superiority of one model is if that model can account for qualitative patterns in the data that are not captured by other models (see section 'Validate (at least) the winning model').

### Parameter fits

Many modeling papers involve fitting parameters to behavioral data. In some cases this is the main point of the paper, for example to show that parameter values differ between groups or treatments, in other cases parameter fitting is secondary to model comparison. In all cases, we recommend reporting the fit parameter values in as transparent a way as possible (i.e. more than just the means and standard errors).

#### Report distributions of parameter values

The simplest way to report parameter fits is to plot a distribution of all fit parameter values, for example in the form of a histogram (e.g. Figure S1 in Wilson et al., 2013 and Nassar et al., 2018) or a cloud of points (e.g. Figure 5 in Huys et al., 2011). This gives a great sense of the variability in each parameter across the population and can also illustrate problems with fitting. For example, if a large number of fit parameters are clustered around the upper and lower bounds, this may indicate a problem with the model.

#### Plot pairwise correlations between fit parameter values

A deeper understanding of the relationships *between* fit parameters can be obtained by making scatter plots of the pairwise correlations between parameters. As with histograms of individual parameters, this approach gives a sense of the distribution of parameters, and can provide evidence of problems with the model; for example, if two parameters trade off against one another, it is a sign that these parameters may be unidentifiable in the experiment.

#### Report parameter recovery

Finally, all parameter fit analyses should sit on the shoulders of a comprehensive parameter recovery analysis with simulated data. If parameters cannot be recovered in the ideal case of simulated data, there is little that they can tell us about real behavior.

### Share your data and code!

The most direct way to communicate your results is to share the data and code. This approach encourages transparency and ensures that others can see *exactly* what you did. Sharing data and code also allows others to extend your analyses easily, by applying it to their own data or adding new models into the mix.

Ideally the data you share should be the raw data for the experiment, with minimal or no preprocessing (apart from the removal of identifying information). The code you share should reproduce all steps in your analysis, including any preprocessing/outlier exclusion you may have performed and generating all of the main and supplementary figures in the paper. In a perfect world, both data and code would be shared publicly on sites such as GitHub, DataVerse and so on. However, this is not always possible, for example, if the data come from collaborators who do not agree to data sharing, or if further analyses are planned using the same data set. In this case, we recommend having a clean set of ‘shareable’ code (and hopefully data too) that can be sent via email upon request.

### Should you always report all of your modeling results?

Finally, if you are using an established model, it can be tempting to skip many of the steps outlined above and report only the most exciting results. This temptation can be even greater if you are using code developed by someone else that, perhaps, you do not fully understand. In our opinion, taking shortcuts like this is dangerous. For one thing, your experiment or population may be different and the model may perform differently in this regime. For another, quite often ‘established’ models (in the sense that they have been published before), have not been validated in a systematic way. More generally, as with any research technique, when using computational modeling you need to demonstrate that you are applying the method correctly, and the that steps we outline here can help. In conclusion, even if developing the model is not the central point of your paper, you should report all of your modeling results.

## What now?

### Looping back

A modeler’s work is never done. To paraphrase George Box, there are no correct models, there are only useful models (Box, 1979). To make your model more useful, there are a number of next steps to consider to test whether your model really does describe a process in the mind.

#### Improve the model to account for discrepancies with your existing data set

Model fits are never perfect and, even in the best cases, there are often small discrepancies with actual data. The simplest next step is to try to address these discrepancies by improving the model, either by including additional factors (such as side bias or lapse rates) or by devising new models entirely.

#### Use your model to make predictions

The best models don’t just explain data in one experiment, they *predict* data in completely new situations. If your model does not easily generalize to new situations, try to understand why that is and how it could be adjusted to be more general. If your model does generalize, test its predictions against new data — either data you collect yourself from a new experiment or data from other studies that (hopefully) have been shared online.

### Using advanced techniques

Another potential next step is to use more powerful modeling techniques. We focused here on the simplest techniques (maximum likelihood estimation and model comparison by BIC) because of their accessibility to beginners, and because most of the advice we give here generalizes to more advanced techniques. In particular, no matter how advanced the modeling technique used, validation is essential (Palminteri et al., 2017; Nassar and Frank, 2016; Huys, 2017). Nevertheless, the simple methods described here have known limitations. More advanced techniques attempt to remedy them, but come with their own pitfalls. A complete review of these advanced techniques is beyond the scope of this paper; instead we provide pointers to a few of the most interesting techniques for the ambitious reader to pursue.

#### Compute maximum a posteriori (MAP) parameter values

Perhaps the simplest step for improving parameter estimates is to include prior information about parameter values. When combined with the likelihood, these priors allow us to compute the posterior, which we can use to find the maximum a posteriori (MAP) parameter values. Although they are still point estimates, with good priors, MAP parameters can be more accurate than parameters estimated with maximum likelihood approaches (Gershman, 2016; Daw, 2011), although when the priors are bad, this method has problems of its own (Katahira, 2016).

#### Approximate the full posterior by sampling

Point estimates of model parameters, such as those obtained with MLE or MAP, lose interesting information about uncertainty over the parameter distribution. Sampling approaches (such as Markov Chain Monte Carlo or MCMC) provide this richer information; furthermore, they allow modelers to investigate more complex assumptions. For example, hierarchical Bayesian approaches make it possible to fit all participants simultaneously, integrating assumptions about their dependence (e.g. one single group, multiple groups, effects of covariates of interest such as age and so on; Lee, 2011; Lee and Wagenmakers, 2014; Wiecki et al., 2013).

#### Advanced optimizers and approximate likelihood

Some models have intractable likelihoods, for example if the choice state has too many dimensions, as in continuous movements, or if the model included unobservable choices. There exist methods to approximate likelihoods to relate them quantitatively to data, such as the ABC method (Turner and Sederberg, 2012; Sunnåker et al., 2013). There are also advanced methods for finding best fit parameters in a sample-efficient manner when computing the likelihood is expensive (Acerbi and Ji, 2017; Acerbi, 2018).

#### Model selection

Bayesian model selection provides less biased, statistically more accurate ways of identifying which model is best at the group level (Rigoux et al., 2014). This may be particularly important when comparing model selection between groups, for example between patients and controls (Piray et al., 2018).

#### Incorporating other types of data

We focused on modeling a single type of observable data, choices. However, there is a rich literature on fitting models to other measurements, such as reaction times (Ratcliff, 1978; Ratcliff and Rouder, 1998), but also to eye movements and neural data (Turner et al., 2016). Furthermore, fitting more than one measurement at a time provides additional constraints to the model, and as such may provide better fit (Ballard and McClure, 2019). However, fitting additional data can increase the complexity of the model-fitting process and additional care must be taken to determine exactly how different types of data should be combined (Viejo et al., 2015).

## Epilogue

Our goal for this paper was to offer practical advice, for beginners as well as seasoned researchers, on the computational modeling of behavioral data. To this end, we offered guidance on how to generate models, simulate models, fit models, compare models, validate models, and extract latent variables from models to compare with physiological data. We have talked about how to avoid common pitfalls and misinterpretations that can arise with computational modeling, and lingered, quite deliberately, on the importance of good experimental design. Many of these lessons were lessons we learned the hard way, by actually making these mistakes for ourselves over a combined 20+ years in the field. By following these steps, we hope that you will avoid some of the errors that slowed our own research, and that the overall quality of computational modeling in behavioral science will improve.

## Appendix 1

The theory of model fitting Formally, the goal of model fitting is to estimate the parameters, ${𝜽}_{m}$, for each model, m, that best fit the behavioral data. To do this, we take a Bayesian approach and aim to compute (or at least approximate) the posterior distribution over the parameters given the data, $p(\theta_{m}|d_{1:T},m)$. By Bayes’ rule we can write this as

(12) $$p(\theta_{m}|d_{1:T},m)=\frac{p(d_{1:T}|\theta_{m},m)p(\theta_{m}|m)}{p(d_{1:T}|m)}$$

where $p(\theta_{m}|m)$ is the prior on the parameters, ${𝜽}_{m}$; $p(d_{1:T}|\theta_{m},m)$ is the likelihood of the data given the parameters; and the normalization constant, $p\,\left(d_{1:T}|m\right)$, is the probability of the data given the model (which is also known as the marginal likelihood [Lee and Wagenmakers, 2014], more on this below). Because the probabilities tend to be small, it is often easier to work with the log of these quantities

(13) $$\log p\,\left({𝜽}_{m}|d_{1:T},m\right)=\underset{\text{log likelihood}}{\underbrace{\log p\,\left(d_{1:T}|{𝜽}_{m},m\right)}}+\log p\,\left({𝜽}_{m}|m\right)-\log p\,\left(d_{1:T}|m\right)$$

The log-likelihood often gets its own symbol, $L\,L=\log p\,\left(d_{1:T}|{𝜽}_{m},m\right)$, and can be written

(14) $$\log p\,\left(d_{1:T}|{𝜽}_{m},m\right)=\log\left(\prod_{t=1}^{T}p\,\left(c_{t}|d_{1:t-1},s_{t},{𝜽}_{m},m\right)\right)=\sum_{t=1}^{T}\log p\,\left(c_{t}|d_{1:t-1},s_{t},{𝜽}_{m},m\right)$$

where $p\,\left(c_{t}|d_{1:t-1},s_{t},{𝜽}_{m},m\right)$ is the probability of each individual choice given the parameters of the model, which is at the heart of the definition of each model (for example in Equations 1–7). In a perfect world, we would evaluate the log-posterior, $\log p\,\left({𝜽}_{m}|d_{1:T},m\right)$, exactly, but this can be difficult to compute and unwieldy to report. Instead, we must approximate it. This can be done using sampling approaches such as Markov Chain Monte Carlo approaches (Lee and Wagenmakers, 2014), which approximate the full posterior with a set of samples. Another approach is to report a point estimate for the parameters such as the maximum of the log-posterior (the maximum a posteriori [MAP] estimate), or the maximum of the log-likelihood (the maximum likelihood estimate [MLE]). (Note that the log transformation does not change the location of the maximum, so the maximum of the log-likelihood occurs at the same value of ${𝜽}_{m}$ as the maximum of the likelihood.)

(15) $$\begin{array}{cc} {\hat{𝜽}}_{m}^{M\,A\,P} & =\underset{{𝜽}_{m}}{\arg\,\max}\log p\,\left({𝜽}_{m}|d_{1:T},m\right)\\ {\hat{𝜽}}_{m}^{M\,L\,E} & =\underset{{𝜽}_{m}}{\arg\,\max}\log p\,\left(d_{1:T}|{𝜽}_{m},m\right) \end{array}$$

Note that with a uniform prior on ${𝜽}_{m}$, these two estimates coincide. These approaches for estimating parameter values each have different strengths and weaknesses. The MCMC approach is the most principled as, with enough samples, it gives a good approximation of the posterior distribution over each parameter value. This approach also gracefully handles small data sets and allows us to combine data from different subjects in a rigorous manner. Despite these advantages, the MCMC approach is more complex (especially for beginners) and can be slow to implement. On the other hand, point estimates such as the MAP and MLE parameter values are much quicker to compute and often give similar answers to the MCMC approach when the amount of data is large (which is often the case when dealing with young and healthy populations). For this reason, we focus our discussion on the point estimate approaches, focusing in particular on maximum likelihood estimation.

## Appendix 2

The theory of model comparison In model comparison, our goal is to figure out which model of a set of possible models is most likely to have generated the data. To do this, we compute (or at least try to estimate) the probability that model m generated the data, $p\,\left(m|d_{1:T}\right)$. Note that this is the normalization constant from Equation 12. As with parameter recovery, this probability is difficult to compute directly and so we turn to Bayes’ rule and write

(16) $$\begin{array}{cc} p\,\left(m|d_{1:T}\right) & \propto p\,\left(d_{1:T}|m\right)\,p\,\left(m\right)\\ & =\int 𝑑{𝜽}_{m}\,p\,\left(d_{1:T}|{𝜽}_{m},m\right)\,p\,\left({𝜽}_{m}|m\right)\,p\,\left(m\right) \end{array}$$

where p⁢(m) is the prior probability that model m is the correct model and $p\,\left(d_{1:T}|m\right)$ is the likelihood of the data given the model. In most cases, p⁢(m) is assumed to be constant and so we can focus entirely on the likelihood, $p\,\left(d_{1:T}|m\right)$. As before, it is easier to handle the log of this quantity which is known as the marginal likelihood (Lee and Wagenmakers, 2014) or Bayesian evidence, $E_{m}$ (Kass and Raftery, 1995), for model m. More explicitly

(17) $$E_{m}=\log p\,\left(d_{1:T}|m\right)=\log\,\int 𝑑{𝜽}_{m}\,p\,\left(d_{1:T}|{𝜽}_{m},m\right)\,p\,\left({𝜽}_{m}|m\right)$$

If we can compute $E_{m}$ for each model, then the model with the largest evidence is most likely to have generated the data. Note that by integrating over the parameter space, the Bayesian evidence implicitly penalizes free parameters. This is because, the more free parameters, the larger the size of the space over which we integrate and, consequently, the smaller $p\,\left({𝜽}_{m}|m\right)$ is for any given parameter setting. Thus, unless the model predicts the data well for all parameter settings, it pays a price for each additional free parameter. This idea, that simpler models should be favored over more complex models if they both explain the data equally well, is known as Occam’s razor (see Chapter 28 in MacKay, 2003). Unfortunately, because it involves computing an integral over all possible parameter settings, computing the marginal likelihood exactly is usually impossible. There are several methods for approximating the integral based on either replacing it with a sum over a subset of points (Wagenmakers et al., 2010; Lee and Wagenmakers, 2014) or replacing it with an approximation around either the MAP or MLE estimates of the parameters. The latter approach is the most common and three particular forms are used: the Bayes Information Criterion (BIC) (Schwarz, 1978), Akaike information criterion (AIC) (Akaike, 1974) and the Laplace approximation (Kass and Raftery, 1995). Here, we will focus on BIC which is an estimate based around the maximum likelihood estimate of the parameters, ${\hat{𝜽}}_{m}^{M\,L\,E}$,

(18) $$B\,I\,C=-2\,\log\hat{ℒ}+k_{m}\,\log\left(T\right)\approx -2\,\log E_{m}$$

where $k_{m}$ is the number of parameters in model m and $\hat{ℒ}$ is the value of the log-likelihood at ${\hat{𝜽}}_{m}^{M\,L\,E}$. Finally, we have found it useful to report the results of model comparison in terms of the likelihood-per-trial L⁢P⁢T, which can be thought of as the ‘average’ probability with which the model predicts each choice,

$$L\,P\,T=\exp\left(\frac{E_{m}}{T}\right)$$

## Appendix 3

Computing the inversion matrix from the confusion matrix In section 'Can you arbitrate between different models?' we introduced the inversion matrix, p⁢(simulated model|fit model), as the probability that data best fit by one model were actually generated from another model. As shown below, this can be readily computed from the confusion matrix, p⁢(fit model|simulated model), by Bayes rule. Abbreviating ‘simulated model’ with ‘sim’ and ‘fit model’ with ‘fit’ we have

(20) $$p\,\left(\text{sim}|\text{fit}\right)=\frac{p\,\left(\text{fit}|\text{sim}\right)\,p\,\left(\text{sim}\right)}{\sum_{\text{sim}}p\,\left(\text{fit}|\text{sim}\right)\,p\,\left(\text{sim}\right)}$$

For a uniform prior on models, computing the inversion matrix amounts to renormalizing the confusion matrix over the simulated models.

## Appendix 4

Working memory model used for local minima example The model and experimental designs used in Box 3—figure 1 are a simplified version of those in Collins and Frank (2012). In short, the experiment attempts to parse out working memory contributions to reinforcement learning by having participants and agents learn stimulus-action contingencies from deterministic feedback, with a different number of stimuli n⁢s being learned in parallel in different blocks. This manipulation targets WM load and isolates WM contributions; see Collins and Frank (2012) for details. The simplified model assumes a mixture of a classic RL component (with parameters α and β) and a working memory component with perfect one-shot learning. The mixture is controled by parameter ρ, capturing the prior willingness to use working memory vs. RL, and capacity parameter K, which scales the mixture weight in proportion to the proportion of stimuli that may be held in working memory: $\min\left(1,\frac{K}{n\,s}\right)$. The original model assumes additional dynamics for the working memory policy and working memory vs. RL weights that render the model more identifiable (Collins and Frank, 2012).

## Appendix 5

Model validation example In this example, we imagine a deterministic stimulus-action learning task in which subjects are presented with one of three stimuli ($s_{1}$, $s_{2}$, and $s_{3}$), which instruct the subject which of three actions ($a_{1}$, $a_{2}$, and $a_{3}$) will be rewarded when chosen. The two models that we consider are both reinforcement learning agents. The first, a ‘blind’ agent, does not see the stimulus at all and learns only about the value of the three different actions, i.e. $Q\,\left(a_{i}\right)$, regardless of the stimulus. The second, a ‘state-based’ agent, observes the stimulus and learns a value for each action that can be different for each stimulus, i.e. $Q\,\left(a_{i},s_{i}\right)$. Learning in both models occurs via a Rescorla-Wagner rule with different learning rates for positive and negative prediction errors. Thus for the blind agent, the values update according to

(21) $$Q\,\left(a_{t}\right)\leftarrow \left\{ \begin{array}{cc} Q\,\left(a_{t}\right)+\alpha_{P}\,\left(r_{t}-Q\,\left(a_{t}\right)\right) & \text{if }\,\left(r_{t}-Q\,\left(a_{t}\right)\right)>0\\ Q\,\left(a_{t}\right)+\alpha_{N}\,\left(r_{t}-Q\,\left(a_{t}\right)\right) & \text{if }\,\left(r_{t}-Q\,\left(a_{t}\right)\right)<0 \end{array}\right.$$

while for the state-based agent, values update according to

(22) $$Q\,\left(a_{t},s_{t}\right)\leftarrow \left\{ \begin{array}{cc} Q\,\left(a_{t},s_{t}\right)+\alpha_{P}\,\left(r_{t}-Q\,\left(a_{t},s_{t}\right)\right) & \text{if }\,\left(r_{t}-Q\,\left(a_{t},s_{t}\right)\right)>0\\ Q\,\left(a_{t},s_{t}\right)+\alpha_{N}\,\left(r_{t}-Q\,\left(a_{t},s_{t}\right)\right) & \text{if }\,\left(r_{t}-Q\,\left(a_{t},s_{t}\right)\right)<0 \end{array}\right.$$

In both models, these values guide decisions via softmax decision rule with inverse temperature parameter, β. We begin by simulating two different agents: one using the blind algorithm and the other using the state-based approach. Parameters in the models are set such that the learning curves for the two agents are approximately equal (Box 7—figure 1A, blind model: $\alpha_{P}=0.5$, $\alpha_{N}=0$, β=6.5 state-based model: $\alpha_{P}=0.65$, $\alpha_{N}=0$, β=2). In both cases, the agents start from an accuracy of 1/3 and an asymptote at an accuracy of around 2/3 — the blind agent because this is the best it can do, the state-based agent because the softmax parameter is relatively small and hence performance is limited by noise. Next we consider how the state-based model fits behavior from these two different agents. In Box 7—figure 1B, we plot the average likelihood with which the state-based model predicts the actual choices of the blind and state-based agents, that is the average $p\left(c_{t}|d_{1:t-1},{𝜽}_{m},m=\text{state-based}\right)$. As is clear from this figure, the state-based model predicts choices from the blind agent with *higher* likelihood than choices from the state-based agent! Although counter intuitive, this result does not imply that the state-based model is unable to fit its own behavior. Instead, this result reflects the difference in noise (softmax parameters) between the two subjects. The blind RL subject has a high β, implying less noise, allowing the state-based model to fit it quite well. Conversely, the state-based RL subject has a low β, implying more noise, meaning that the behavior is harder to predict even when it is fit with the correct model. That the state-based model fits state-based behavior better than it fits blind behavior is illustrated in Box 7—figure 1C. Here we plot the simulated learning curves of the state-based model using the parameter values that were fit to either the state-based agent or the blind agent. While the fit to the state-based agent generates a learning curve quite similar to that of the subject (compare blue lines in Box 7—figure 1A and C), the state-based fit to the blind agent performs too well (compare yellow lines in panels Box 7—figure 1A and C). Thus the model validation step provides support for the state-based model when it is the correct model of behavior, but rules out the state-based model when the generating model was different.
